# Rapid Shallow Breathing Index Survey, a Predictor of non-Invasive Ventilation Necessity in Patients With Chronic Obstructive Pulmonary Disease Exacerbation: An Analytical Descriptive Prospective Study

**DOI:** 10.5812/ircmj.13326

**Published:** 2014-02-09

**Authors:** Hassan Soleimanpour, Ali Taghizadieh, Rasoul Salimi, Samad EJ Golzari, Ata Mahmoodpoor, Saeid Safari, Robab Mehdizadeh Esfanjani, Yaghoub Heshmat

**Affiliations:** 1 Cardiovascular Research Center, Tabriz University of Medical Sciences, Tabriz, IR Iran; 2 Tuberculosis and Lung Disease Research Center, Tabriz University of Medical Sciences, Tabriz, IR Iran; 3 Students’ Research Committee, Tabriz University of Medical Sciences, Tabriz, IR Iran; 4 Medical Philosophy and History Research Center, Tabriz University of Medical Sciences, Tabriz, IR Iran; 5 Anesthesiology and Critical Care Department, Tabriz University of Medical Sciences, Tabriz, IR Iran; 6 Anesthesiology and Critical Care Department, Iran University of Medical Sciences, Tehran, IR Iran; 7 Neurosciences Research Center, Tabriz University of Medical Sciences, Tabriz, IR Iran

**Keywords:** Noninvasive Ventilation, Blood, APACHE II

## Abstract

**Background:**

Patients with Chronic Obstructive Pulmonary Disease (COPD) are susceptible to respiratory failure which would ultimately lead to their hospitalization. Need to Non-Invasive Ventilation (NIV) is considered as the choice respiratory support in acute respiratory failure and is associated with a beneficial role in patients with COPD exacerbation. Hence, determining patients that would benefit NIV could be of great assistance.

**Objectives:**

We aimed at evaluating the use of Rapid Shallow Breathing Index (a ratio determined by the frequency (f) divided by the tidal volume (VT)) in NIV requirement in COPD patients.

**Patients and Methods:**

In a prospective descriptive study, ninety eight patients over 40 years old with documented COPD exacerbation who were referred to emergency department of Imam Reza hospital, Tabriz, Iran were studied. Rapid Shallow Breathing Index (RSBI), ABG parameters and APACHE II scoring were measured in each patient. Quantitative data were analyzed by Student's t-test and One-way ANOVA and qualitative data were analyzed using chi square (X2). Findings were analyzed with SPSS software version 16.

**Results:**

Patients requiring NIV included 43.9 % of all studied patients. RSBI and APACHE II score with sensitivity of 94.8 %, (cut off point = 110) and 72 % (cut off point = 14) respectively, had high diagnostic sensitivity and also the ability to predict patients requiring NIV. None of ABG parameters solely played a significant role in determining patients requiring NIV.

**Conclusions:**

RSBI and APACHE II score in patients with COPD exacerbation are of the ability to predict NIV requirement, as a predicting factor of Non-Invasive Ventilation requirement.

## 1. Background

COPD is characterized by an airflow limitation which is not fully reversible (1). At the moment, COPD is the fourth leading cause of morbidity and mortality throughout the world and is predicted to be known as the fifth cause of disability by 2020 (2, 3). Patients with COPD are susceptible to respiratory failure which would ultimately lead to their hospitalization.

One third to one fifth of the hospitalized patients suffering from secondary acute hypercapnic respiratory failure due to COPD exacerbation would expire despite being mechanically ventilated (4-8). Conventional treatments (bronchodilators, corticosteroids, antibiotics, and controlled oxygen) are mostly based on providing constant and sufficient oxygenation and treating the cause of disease exacerbation as well. However, the process of intubation and ventilation support in these patients is associated with an increased rate of complications and it would be difficult to wean these patients from ventilator (9, 10). Considering the complications associated with mechanical ventilation in patients with acute respiratory failure or COPD, researches have shown that Non-Invasive ventilation (NIV) could aid ventilation by reducing respiratory work; therefore reducing intubation, morbidity and mortality rates in these patients (11, 12). To determine indications of requiring NIV in patients suffering respiratory failure, available guidelines are based on ABG findings in these patients which eventually would expose them to an invasive procedure; therefore, providing a non-invasive predictive factor necessitating NIV could be of great importance. Few studies have compared proper non-invasive indices predicting NIV requirement in patients with COPD. The rapid shallow breathing index (RSBI), being first introduced by Yang and Tobin in 1991, is a ratio determined by the frequency (f) divided by the tidal volume (VT). Healthcare professionals have been using this ratio successfully in most mechanical ventilation weaning protocols; as RSBI < 105 is considered as a criterion for weaning to extubation (13).

## 2. Objectives

In this research, we hypothesized using Rapid Shallow Breathing Index (RSBI) conversely as a predictor of NIV requirement in patients with documented COPD exacerbation.

## 3. Methods and Materials

This descriptive-prospective study was carried out in emergency ward of Imam Reza Hospital, Tabriz, Iran (14). Imam Reza hospital is a general and public 300-bed educational referral hospital with 24 in-patient wards. This study was approved by the Ethics Committee of “Tabriz University of Medical Sciences” and registered under the Code Number 883. However, all aspects of the present study plan were explained to patients and then we obtained their written consent, including consent to participate in the study and consent to publish, where appropriate. Regarding the sample size evaluation, as we could not find any similar study in the literature review, we performed the study in a 6-month period (2011 March to 2011 August) and included all COPD patients who had the inclusion criteria in this period. Patient collection was performed from 8 a.m. until 4 p.m. seven days a week while no sample collection was performed in the evening or night shifts. The inclusion criteria were all the patients over 40 years old, COPD Exacerbation, History of smoking at least twenty pack per year.

Note: Smoking contributes to the incidence of COPD immensely (90 % of the cases with COPD are caused by smoking); however COPD is rare before the age of 40 and many years are required for smoking to decrease FEV1 rapidly leading to COPD and in patients younger than 40 with COPD signs, other diagnoses should also be born in mind. Therefore, in the current study only were COPD patients older than 40 included (15). The exclusion criteria: Respiratory arrest, cardiovascular instability, lack of patient cooperation, upper airway obstruction, possibility of increased aspiration risk, morbid obesity, increase in secretions, recent facial trauma or gastroesophageal surgery, impaired mask fixation, nasopharyngeal abnormalities, craniofacial trauma, lack of patient consent. For ABG analysis RAPID Point 340/350 Blood Gas Systems (Siemens, Germany) and for pulse oximetry, Alborz B7 (Saadat, Iran) were used. All instruments were calibrated prior to use. To determine the need for NIV, in all patients, ABG and simultaneous RSBI were measured using ventilator model “Vector ST40” with BLPAP mode EPAP = 5 cm H_2_O, IPAP = 10 cm H_2_O. Furthermore, medical treatments (intravenous corticosteroid, salbutamol spray, atrovent spray, antibiotics, and oxygen therapy) were administered. Duration and method of oxygen therapy was as following for all patients.

Indications for commencement of non-invasive ventilation (NIV) included exacerbation of COPD with PH < 7.35 and PaCO_2_ > 45mmHg or PaO_2_ < 60mmHg (SpO_2_ < 9 0%) despite receiving Max FIO_2_ of 60%. Titrated Oxygen therapy was initiated using Venturi mask at FIO_2_ of 24% to 60% for all patients based on pulse oximetry every 20 minutes to achieve SpO_2_ > 90% (for maximum of two hours). In case of not achieving SPO_2_ > 90% despite receiving FIO_2_ > 60%, patients underwent NIV. After administering the above-mentioned treatments and oxygen therapy for two hours, required variables on admission, one hour and two hours after treatment were measured; ultimately patients were divided into two groups of requiring non-invasive ventilation (group I) and not requiring non-invasive ventilation (group II). Patients requiring non-invasive ventilation were hospitalized after consulting with a pulmonologist and patients who were not hospitalized or did not require non-invasive ventilation, depending on clinical condition, were discharged from emergency service or referred to pulmonary subspecialty clinic. Findings obtained from the measured RSBI and ABG of the patients in three stages and also APACHE II score for two groups were analyzed using SPSS16 software. For statistical study, descriptive statistical analysis was used (frequency, percentage, mean ± standard deviation). Normal data distribution was evaluated by Kolmogorov-Smirnov test. Quantitative data, if required, were analyzed by Student’s t-test or Mann-Whithney U test, also Repeated Measurements Analysis and qualitative data were analyzed by Chi-square test (X^2^). Logistic Regression tests were used for determining effective factors on patients requiring NIV. In addition, ROC was used to determine sensitivity of different variables in predicting NIV requirement. In this study, P value less than 0.05 was considered significant.

## 4. Results

In the present study, 98 patients including 64 males and 34 females were examined. The mean age of the patients was 68.43 ± 10.29 years with the median of 68.5 years. The youngest and the oldest patients were 42 and 89 years, respectively. The mean age of patients was 68.69 ± 10.94 and 68.23 ± 9.84 years in groups requiring NIV (group I) and not requiring NIV (group II), respectively. No significant difference was observed between both groups in this regard (P = 0.82). Furthermore, there was no significant difference between two groups considering sex distribution (P = 0.83). Among vital signs measured in three stages (on admission, an hour and two hours after admission), respiration rate and heart rate in each three stage were significantly high in group requiring NIV. Table 1 illustrates the findings of ABG analysis between two groups. As it can be seen, findings of ABG in patients not requiring NIV are considerably better than group I. Table 2 presents respiratory findings in two groups. As it can be seen, respiratory findings in patients not requiring NIV are also significantly better than group I.

**Table 1.** ABG Analysis Findings Between Two Groups ^a^

	Group I (Requiring NIV)	Group II (Not Requiring NIV)	P value Within Group	P value Between Group
**PH at admission**	7.24 ± 0.05	7.34 ± 0.05	< 0.0001	< 0.0001
**PH 60 minutes later**	7.26 ± 0.04	7.34 ± 0.04	< 0.0001	< 0.0001
**PH 120 minutes later**	7.27 ± 0.03	7.36 ± 0.02	< 0.0001	< 0.0001
**PaO_2_** ** at admission(mmHg)**	43.86 ± 6.37	49.46 ± 5.79	< 0.0001	< 0.0001
**PaO_2_** ** 60 minutes later (mmHg)**	52.38 ± 4.04	55.02 ± 4.78	< 0.0001	< 0.0001
**PaO_2_** ** 120 minutes later (mmHg)**	55.72 ± 3.51	62.82 ± 2.06	< 0.0001	< 0.0001
**PaCO_2_** ** at admission (mmHg)**	62.73 ± 15.27	49.26 ± 12.26	0.009	< 0.0001
**PaCO_2_** ** 60 minutes later (mmHg)**	60.84 ± 14.00	45.76 ± 12.05	0.009	< 0.0001
**PaCO_2_** ** 120 minutes later (mmHg)**	58.99 ± 11.18	46.70 ± 9.54	0.009	< 0.0001
**BE at admission**	-0.19 ± 0.74	0.69 ± 0.54	0.136	0.42
**BE 60 minutes later**	-0.21 ± 0.60	- 0.67 ± 0.58	0.136	0.42
**BE 120 minutes later**	-0.24 ± 0.53	0.90 ± 0.44	0.136	0.42
**HCO_3_** ** at admission (mEq /L)**	28.06 ± 5.12	26.20 ± 4.95	0.065	0.007
**HCO_3_** ** 60 minutes later (mEq /L)**	27.36 ± 4.37	25.39 ± 4.54	0.065	0.007
**HCO_3_** ** 120 minutes later (mEq /L)**	27.73 ± 4.67	24.53 ± 5.35	0.065	0.007
**SpO_2_** ** at admission (%)**	62.48 ± 9.68	70.29 ± 12.82	< 0.0001	< 0.0001
**SpO_2_** ** 60 minutes later (%)**	77.09 ± 6.69	82.78 ± 6.25	< 0.0001	< 0.0001
**SpO_2_** ** 120 minutes later (%)**	84.10 ± 5.06	91.47 ± 1.47	< 0.0001	< 0.0001

^a^ Abbreviations: NIV: Non-Invasive Ventilation

**Table 2.** Respiratory Findings in Both Groups ^a^

	Group I (Requiring NIV)	Group II (Not Requiring NIV)	P value Within Group	P value Between Group
**TV at admission**	-	-	< 0.0001	< 0.0001
Percentile 25	176	247	-	-
Percentile 50	202	277	-	-
Percentile 75	221	312	-	-
**TV 60 minutes later**	-	-	< 0.0001	< 0.0001
Percentile 25	185	257	-	-
Percentile 50	204	288	-	-
Percentile 75	228	338	-	-
**TV 120 minutes later**	-	-	< 0.0001	< 0.0001
Percentile 25	188	261	-	-
Percentile 50	188	296	-	-
Percentile 75	231	351	-	-
**RSBI at admission**	-	-	< 0.0001	< 0.0001
Percentile 25	132.1	63.1	-	-
Percentile 50	152.3	73	-	-
Percentile 75	186	96.1	-	-
**RSBI 60 minutes later**	-	-	< 0.0001	< 0.0001
Percentile 25	114	67.3	-	-
Percentile 50	126.5	77.9	-	-
Percentile 75	145.8	89.4	-	-
**RSBI 120 minutes later**	-	-	< 0.0001	< 0.0001
Percentile 25	106.3	56.7	-	-
Percentile 50	112.1	70.1	-	-
Percentile 75	121.3	80.7	-	-
**MV at admission**	-	-	0.81	0.04
Percentile 25	4.44	5.54	-	-
Percentile 50	6.24	6.86	-	-
Percentile 75	7.39	7.8	-	-
**MV 60 minutes later**	-	-	0.81	0.04
Percentile 25	4.35	5.45	-	-
Percentile 50	5.62	6.62	-	-
Percentile 75	6.66	7.5	-	-
**MV 120 minutes later**	-	-	0.81	0.04
Percentile 25	4.46	5.17	-	-
Percentile 50	5.42	6.21	-	-
Percentile 75	6.15	7.19	-	-

^a^ Abbreviations: NIV: Non-Invasive Ventilation; TV: Tidal Volume; RSBI: Rapid Shallow Breathing Index; MV: Minute ventilation

**Figure 1.**
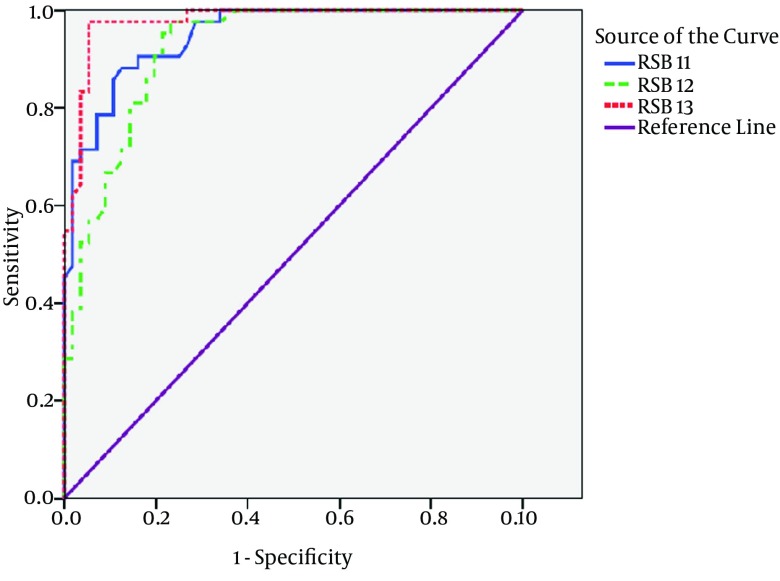
ROC Curve for RSBI Measurement at Three Stage of Measurement

None of the variables resulted from ABG analysis based on Logistic regression model revealed any significant role as predicting factor of invasive ventilation necessity in patients with COPD exacerbation. Using Logistic Regression statistical tests to evaluate the predictive value of ABG, Tidal volume (TV), Minute ventilation (MV) and RSBI variables for non-invasive ventilation necessity in patients with COPD exacerbation revealed that RSBI prior to treatment and an hour and two hours subsequent to treatment, in addition to possessing high diagnostic sensitivity in patients requiring NIV, has a significant predictive ability on admission [Odd’s ratio equal to (CI 1.04-1.09) 1.07 and P < 0.001], an hour [Odd’s ratio equal (CI 1.05-1.1) 1.08 and P < 0.001] and two hours subsequent to treatment [Odd’s ratio was (CI 1.09 - 1.27) 1.1 and P < 0.001] in patients requiring NIV. As at each measured stages (on admission, an hour and two hours subsequent to treatment) evaluated sensitivity ratios were 94.8 %, 92.8 % , 97.7 % and specificity values were 94.8 %, 92.8 % and 97.7 %, respectively. Also Youden’s index (J), defined by: J = maximum {sensitivity (c) + specificity (c) – 1} were 0.96, 0.84 and 0.94, respectively and values for cutoff point were more or equal to 110, 105 and 107, respectively (Figure 1). 

**Figure 2.**
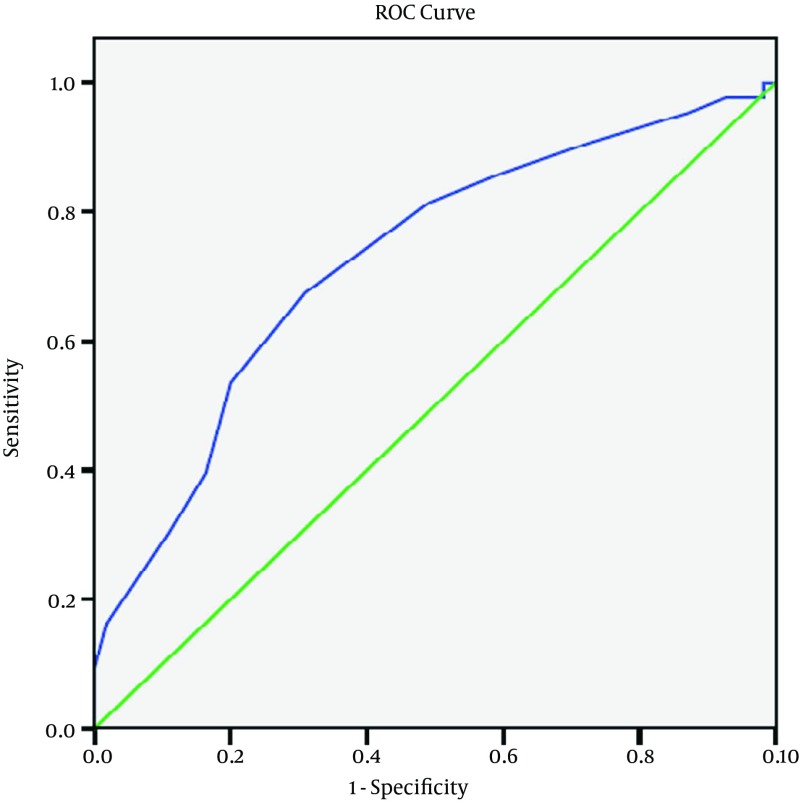
ROC Curve for APACHE II Scoring

In the study in addition to RSBI, APACHE II score (with measured mean scores of 16.67 ± 3.29 and 14.12 ± 3.12 in groups I and II respectively) was of high sensitivity [Odd’s ratio with (CI 1.5 - 1.1) 1.29 and P = 0.001] with sensitivity of 72 %, specificity of 72 % and cut of point of 14. And based on Regression model, APACHE II score had a significant predicting role for non-invasive ventilation necessity in patients with COPD exacerbation (Figure 2). Eventually after two hours administration of oxygen, 43 patients (43.9 %) were placed in group requiring NIV and 55 patients (56.1 %) in group not requiring NIV.

## 5. Discussion

To determine indications of NIV, different criteria have been stated most of which necessitate ABG analysis for definite indication of NIV requirement (16). Indications of NIV commencement include: COPD exacerbation, moreover PH < 7.32 and PaCO_2_ > 45 mmHg or PaO_2_ < 60mmHg despite receiving oxygen with Max FIO_2_ of 60% (12). Very few studies has been carried out to eliminate invasive interventions for determining NIV indication including Crawford’s study in which different parameters have been studied for determining the index of NIV requirement such as: RSBI, PH, Lactate, MV, Carbon Dioxide production (VCO_2_), End-Tidal CO_2_ (ETCO2), Air way dead space and APACHE II criterion. In this study using the Receiver Operating Characteristic (ROC) value revealed that RSBI more or equal to 120 was associated with the highest sensitivity and specificity for determining the need for non-invasive ventilation and it was consequently declared as a non-invasive index for evaluating the NIV requirement in patients with acute respiratory failure in emergency department (17). However, in the study of Lin et al. RSBI failed to be considered as a good predicting factor of successful non-invasive ventilation (NIV) intervention in patients with acute respiratory failure. In the study, it was stated that APACHE II score prior to treatment, PImax 30 minutes later, and RR 30 and 60 minutes later were all significantly lower in group not requiring NIV (18). Youshida et al. observed that patients in need of intubation had significantly higher APACHE II scores and lower arterial pH, as APACH II score higher than 17 and respiratory rate above 25 per minute after receiving NIV for an hour were introduced as independent determinants of requiring intubation (19). Our study, compared to other studies, is one of the few studies to have examined APACHE II score and various indices such as ABG and RSBI regarding their predictive ability for NIV requirement in patients with COPD exacerbation. Furthermore, more sample size of this study compared to Crawford’s (98 to 61) and also following-up the predictive ability of RSBI in NIV requirement in the first two hours of treatment are considered as the advantages of this research. On the other hand, the sampling strategy and inability to generalized finding to the target population are considered as the limitations of the present study. In this research RSBI and APACHE II score in patients with COPD exacerbation revealed the ability to predict the need for NIV, as a predicting factor of non-invasive ventilation requirement. It is therefore recommended that APACHE II and RSBI factors be used as predicting factors in non-invasive ventilation requirement in patients with COPD exacerbation.
